# Current incidence, severity, and management of veno-occlusive disease/sinusoidal obstruction syndrome in adult allogeneic HSCT recipients: an EBMT Transplant Complications Working Party study

**DOI:** 10.1038/s41409-023-02077-2

**Published:** 2023-08-12

**Authors:** Tapani Ruutu, Christophe Peczynski, Mohamed Houhou, Emmanuelle Polge, Mohamad Mohty, Nicolaus Kröger, Ivan Moiseev, Olaf Penack, Nina Salooja, Hélène Schoemans, Rafael F. Duarte, Thomas Schroeder, Jakob Passweg, Gerald G. Wulf, Arnold Ganser, Simona Sica, Mutlu Arat, Urpu Salmenniemi, Annoek E. C. Broers, Jean Henri Bourhis, Alessandro Rambaldi, Johan Maertens, Kazimierz Halaburda, Tsila Zuckerman, Hélène Labussière-Wallet, Grzegorz Basak, Christian Koenecke, Zinaida Perić

**Affiliations:** 1 Clinical Research Institute, Helsinki University Hospital and University of Helsinki, Helsinki, Finland; 2 EBMT, Transplant Complications Working Party, Hopital Saint Antoine, Department of Hematology, Paris, France; 3 EBMT, Sorbonne University, Hopital Saint Antoine, Department of Hematology, Paris, France; 4 University Hospital Eppendorf, Hamburg, Germany; 5 RM Gorbacheva Research Institute, Pavlov University, Saint Petersburg, Russian Federation; 6 Medical Clinic, Department of Haematology, Oncology and Tumor Immunology, Charité Universitätsmedizin Berlin, Berlin, Germany; 7 Centre for Haematology, Imperial College, London, UK; 8 Department of Hematology, University Hospitals Leuven and KU Leuven, Leuven, Belgium; 9 Department of Hematology, Hospital Universitario Puerta de Hierro Majadahonda, Madrid, Spain; 10 University Hospital, Department of Hematology and Stem Cell Transplantation, Essen, Germany; 11 University Hospital, Hematology, Basel, Switzerland; 12 University Medicine Goettingen, Department of Haematology and Medical Oncology, Goettingen, Germany; 13 Hannover Medical School, Department of Hematology, Hemostasis, and Oncology, Hannover, Germany; 14 Dipartimento di Diagnostica per Immagini, Radioterapia Oncologica ed Ematologia, Fondazione Policlinico Universitario A. Gemelli IRCCS, Roma, Università Cattolica Sacro Cuore, Rome, Italy; 15 Demiroglu Bilim University, Istanbul Florence Nightingale Hospital, Hematopoietic SCT Unit, Istanbul, Turkey; 16 HUCH Comprehensive Cancer Center, Stem Cell Transplantation Unit, Helsinki, Finland; 17 Department of Hematology, Erasmus MC Cancer Institute, Rotterdam, The Netherlands; 18 Gustave Roussy Cancer Campus, BMT Service, Department of Hematology, Villejuif, France; 19 Department of Oncology and Hematology, University of Milan and Azienda Socio-Sanitaria Territoriale Papa Giovanni XXIII, Bergamo, Italy; 20 University Hospital Gasthuisberg, Department of Hematology, Leuven, Belgium; 21 Institute of Hematology and Transfusion Medicine I, Warsaw, Poland; 22 Rambam Medical Center, Department of Hematology and Bone Marrow Transplantation, Haifa, Israel; 23 Hospital Lyon Sud, Hospices Civils de Lyon, Pierre Bénite, France; 24 Department of Hematology, Transplantation and Internal Medicine, Medical University of Warsaw, Warsaw, Poland; 25 Department of Hematology, Hemostasis, Oncology and Stem Cell Transplantation, Hannover Medical School, Hannover, Germany; 26 Department of Hematology, University Hospital Center Zagreb, Zagreb, Croatia

**Keywords:** Acute myeloid leukaemia, Myelodysplastic syndrome

## Abstract

The current incidence, diagnostic policy, management, and outcome of VOD/SOS at EBMT centers were studied. All centers that had performed allogeneic HSCTs in adult patients within one defined year were invited to the study. Seventy-one centers participated with a total of 2886 allogeneic transplantations and 93 cases of VOD/SOS in 2018. The cumulative incidence of VOD/SOS at day 21 was 1.8% and at day 100 2.4%. Of 67 cases with detailed data, 52 were classical and 15 (22%) late onset (>day 21). According to the EBMT criteria, 65/67 patients had at least two VOD/SOS risk factors. The severity grades were: mild 0, moderate 3, severe 29, very severe 35. Fifty-four patients were treated with defibrotide. VOD/SOS resolved in 58% of the patients, 3/3 with moderate, 22/28 with severe, and 12/33 with very severe grade (*p* < 0.001). By day 100, 57% of the patients were alive; 3/3 with moderate, 22/29 with severe, and 13/35 with very severe VOD/SOS (*p* = 0.002). In conclusion, the incidence of VOD/SOS was low. Severe and very severe grades dominated. Very severe grade predicted poor outcome compared to severe grade further supporting the concept of early diagnosis and treatment to avoid a dismal outcome.

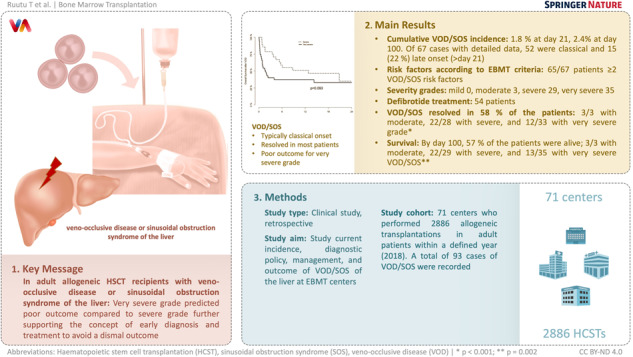

## Introduction

Veno-occlusive disease or sinusoidal obstruction syndrome of the liver (VOD/SOS) is a major, potentially fatal complication of hematopoietic stem cell transplantation, particularly allogeneic transplantation [1]. The toxicity of pretransplant conditioning is thought to play a central role in its pathophysiology, and myeloablative conditioning (MAC) regimens have usually been found to be associated with a higher risk of VOD/SOS compared with reduced intensity regimens (RIC) [1–3]. The reported incidences have varied considerably depending on the patient populations, transplantation methods, and diagnostic criteria. Recently presented estimates of the incidence have been approximately 10–15% in transplantations with MAC, 5% or less in RIC transplantations [1, 2, 4–7], although an 8.8% incidence after RIC transplantations has recently been reported [8]. The incidence may have declined in the recent years [3, 7]. The course of this complication is variable, it can resolve without specific therapeutic measures, but in a large proportion it leads to multiorgan failure with a high mortality rate [2].

Significant developments in the diagnosis and management of this complication have taken place in the recent years. Effective treatment has become available [9]. EBMT (European Society for Blood and Marrow Transplantation) published revised diagnostic criteria for classical and late-onset VOD/SOS, and presented criteria for severity grading [9]. Assessment of severity is important for therapeutic decisions. The severity grading is based on several clinical components and, in addition, on the burden of risk factors.

The Transplant Complications Working Party of the EBMT has carried out a retrospective cohort study to evaluate, applying the EBMT criteria, the current state among EBMT centers regarding the incidence of VOD/SOS, the diagnostic procedures, the prophylaxis and treatment used, the role of the severity grading, and the outcome.

## Methods

All EBMT centers performing allogeneic hematopoietic stem cell transplantations in adult patients were invited to report retrospectively all allogeneic transplantations carried out within one defined year, year 2018, and the number of VOD/SOS cases diagnosed among these patients. They were also asked to fill in an additional (MedC) form of all these VOD/SOS patients including information of risk factors, prophylaxis, diagnostic procedures, management, and outcome not included in the routine EBMT reporting. The invitation to participate was circulated in April 2019, and two reminders were sent.

The diagnostic criteria of VOD/SOS were the EBMT criteria unless otherwise stated. The severity grades were according to the EBMT classification (Table 1). The definition of multiorgan dysfunction/failure (MOD/MOF) is shown in Supplementary Table 1.

**Table 1:** The EBMT severity grading of VOD/SOS [9].

	Mild^a^	Moderate^a^	Severe	Very severe – MOD/MOF^b^
Time since first clinical symptoms of SOS/VOD^c^	>7 days	5–7 days	≤4 days	Any time
Bilirubin (mg/dL)	≥2 and <3	≥3 and <5	≥5 and <8	≥8
Bilirubin (µmol/L)	≥34 and <51	≥51 and <85	≥85 and <136	≥136
Bilirubin kinetics			Doubling within 48 h	
Transaminases	≤2 x normal	>2 and ≤5 × normal	>5 and ≤8 × normal	>8 × normal
Weight increase	<5%	≥5% and <10%	≥5% and <10%	≥10%
Renal function	<1.2 × baseline at transplant	≥1.2 and <1.5 × baseline at transplant	≥1.5 and <2 × baseline at transplant	≥2 × baseline at transplant or other signs of MOD/MOF

Patients belong to the category that fulfills two or more criteria. If patients fulfill two or more criteria in two different categories, they must be classified in the most severe category. Patient´s weight increase ≥ 5% and < 10% is considered by default as a criterion for severe SOS/VOD; however, if patients do not fulfill other criteria for severe SOS/VOD, weight increase ≥ 5% and < 10% is considered as a criterion for moderate SOS/VOD.

*EBMT* European Society for Blood and Marrow Transplantation, *MOD* multiorgan dysfunction, *MOF* multiorgan failure, *SOS* sinusoidal obstruction syndrome, *VOD* veno-occlusive disease.

^a^ln the case of presence of two or more risk factors for SOS/VOD, patients should be in the upper grade.

^b^Patients with multiorgan dysfunction must be classified as very severe.

^c^Time from the date when the first signs/symptoms of SOS/VOD began to appear (retrospectively determined) to the date when the symptoms fulfilled SOS/VOD diagnostic criteria.

### Risk factors

The risk factors analyzed were those listed in the EBMT report presenting the diagnostic and severity criteria [9].

Transplant-related factors: unrelated donor, HLA-mismatched donor, non-T-cell depleted transplant, MAC regimen, oral or high-dose busulfan-based regimen, high-dose TBI-based regimen, second hematopoietic stem cell transplantation.

Patient and disease related factors: older age, Karnofsky score below 90%, metabolic syndrome, female receiving norethisterone, advanced disease (beyond 2nd CR or relapse/refractory), thalassemia, genetic factors (GSTM1 polymorphism, C282Y allele, MTHFR 677CC/1298CC haplotype).

Hepatic related: transaminases >2.5 × ULN, serum bilirubin >1.5 × ULN, cirrhosis, active viral hepatitis, abdominal or hepatic irradiation, previous use of gemtuzumab ozogamicin or inotuzumab ozogamicin, hepatotoxic drugs, iron overload.

According to the EBMT reporting instructions, MAC was defined as a regimen containing either total body irradiation with a dose equal to or greater than 8 Gy, a total dose of oral busulfan greater than 8 mg/kg, or a total dose of intravenous busulfan greater than 6.4 mg/kg [10]. All other regimens were defined as RIC.

In the questionnaire a high dose of busulfan was defined as ≥9.6 mg/kg total dose i.v. or ≥12 mg/kg total dose p.o. Age over 50 years was regarded as older age; the median age of the patients was 48.9 years. Metabolic syndrome was defined according to NCEP ATPIII 2005 [11]. The registering of hepatotoxic drugs for the study was according to the center report.

The data were analyzed at the EBMT Paris data office. The identity of the participating centers and the patients was only known to the data office and not reported to the investigators.

### Statistics

The primary study endpoint was to assess the incidence of VOD/SOS in the first 100 days after allogeneic transplantation. For the estimation of the cumulative incidence of VOD/SOS, death was considered a competing event. A secondary study endpoint was overall survival (OS). Probabilities of OS were calculated using the Kaplan–Meier method. When comparing the outcome of VOD/SOS by severity grade in patients who developed VOD/SOS, the starting point was the date of diagnosis of VOD/SOS.

Statistical analyses were performed with R 4.1.2 software (R Development Core Team, Vienna, Austria) packages.

## Results

Seventy-one centers from 20 countries participated in this study, 24% of all eligible centers (Supplementary Table 2). The total number of allogeneic transplantations in adult patients at these centers in the year 2018 was 2886. Among these patients, 93 cases of VOD/SOS had been diagnosed. Additional VOD/SOS -related data was received of 70 patients; these cases were analyzed in detail. In three of these patients, the EBMT criteria for classical VOD/SOS were not fulfilled and there were no findings indicating late-onset VOD/SOS. In two cases the Seattle criteria had been used. These three patients were excluded, and 67 patients remained for the analysis.

A summary of the patient material and the main results is presented in Table 2.

**Table 2:** Summary of the patient material and the main results.

Participating centers	71
Total number of allogeneic transplantations in 2018	2886
Number of patients with VOD/SOS diagnosis in 2018	93
Cumulative VOD/SOS incidence at 21 days	1.8 (95% CI 1.4–2.4) %
Cumulative VOD/SOS incidence at 100 days	2.4 (95% CI 1.9–3.0) %
Patients with VOD/SOS data for detailed analysis	67
VOD/SOS prophylaxis given	40/67 (60%)
Severity of VOD/SOS according to EBMT criteria	
Moderate	3
Severe	29
Very severe	35
Late onset of VOD/SOS ( > 21 days)	15/67 (22%)
VOD/SOS-targeted treatment given	60/67 (90%)
Defibrotide	54
Resolution of VOD/SOS	37/64 (58%)
Severe grade	22/28 (79%)
Very severe grade	12/33 (36%)
Survival at 100 days post transplant	38/67 (57%)
Severe grade	22/29 (76%)
Very severe grade	13/35 (37%)

### Incidence

The cumulative incidence of VOD/SOS at day 21 was 1.8 (95% CI 1.4–2.4) % and at day 100 2.4 (1.9–3.0) %. As the details of the diagnostic criteria were not available for all patients, this incidence analysis includes all 93 patients with reported VOD/SOS, independent of the criteria used.

### Risk factors

All VOD/SOS patients had risk factors. Two patients had one risk factor, four patients 2, eight patients 3, eight patients 4, and forty-five more than 4 risk factors. The median number of risk factors was 5, range 1–8.

### Prophylaxis

Forty patients (60%) had received VOD/SOS prophylaxis. Defibrotide had been given to 7 patients, either alone (4) or together with ursodeoxycholic acid (3) and/or heparin (1). Ursodeoxycholic acid had been given to 22 patients, heparin to 15 patients either alone (9 patients) or in combination with ursodeoxycholic acid (5) or defibrotide (1).

### Conditioning

The pretransplant conditioning given to the patients who later developed VOD/SOS was MAC in 51.5% and RIC in 48.5% of the cases; in one case the intensity was lacking. Among the whole population of 2886 patients transplantated during the time of the study at the participating centers the conditioning was MAC in 51.9% and RIC in 48.1%.

In the whole patient population, the cumulative incidence of VOD/SOS at 100 days among the patients given MAC was 2.4% and among those given RIC 2.3%.

### Diagnosis of VOD/SOS

**Fig. 1:**
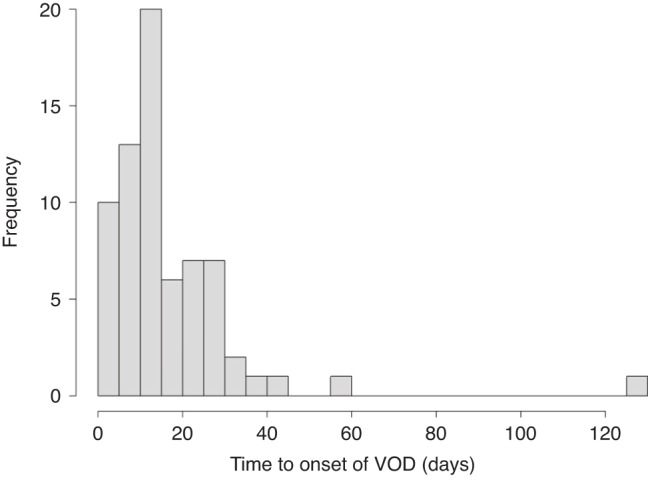
Timing of the onset of VOD/SOS, days post transplantation.

Classical VOD/SOS according to the EBMT (Baltimore) criteria (in the first 21 days after HSCT) was diagnosed in 52 patients, late-onset VOD/SOS (>21 days) according to the EBMT criteria in 15 patients. The timing of the onset of VOD/SOS is shown in Fig. 1.

According to the EBMT criteria, late-onset VOD/SOS can be diagnosed in three situations: 1. The findings of classical VOD/SOS occur after day 21; 2. VOD/SOS is proven histopathologically; or 3. two or more of the following findings are present: bilirubin ≥34 µmol/L, painful hepatomegaly, weight gain >5%, ascites, and in addition there is hemodynamical or/and ultrasound evidence of VOD/SOS. In all but one late-onset case, the criteria of classical VOD/SOS were fulfilled after day 21. In the remaining case the diagnosis was based on the third alternative. Histological evidence for VOD/SOS was obtained in four cases but the diagnosis of late-onset VOD/SOS was not based solely on this criterion in any case.

Overall, in classical and late-onset VOD/SOS, imaging was used to support the diagnosis in 59 of the 67 patients. Ultrasound was used in 58 and CT in 6 cases. A decrease in velocity or reversal of portal flow was demonstrated in 12 patients. Hepatic venous pressure gradient was measured in 3 cases. Liver biopsy was carried out in 5 cases.

### Severity grading

Table 1 shows the EBMT severity grading. In the present patient population, the severity grades of the 67 cases of VOD/SOS were: mild 0, moderate 3, severe 29, and very severe 35. It should be noted that according to the grading patients with mild or moderate VOD/SOS and two or more risk factors must be graded one grade higher. All but two patients had two or more risk factors, and the two with only one risk factor had already originally severe grade VOD/SOS. Therefore in all the three patients with originally mild VOD/SOS the grade was revised to moderate, and all 13 moderate cases became severe.

The grade was classified as very severe in all but one patient based on multiorgan dysfunction/ failure (MOD/MOF). In the remaining case, the criteria were high bilirubin and >10% weight increase.

### Treatment

Sixty of the 67 patients received VOD/SOS-targeted treatment. This was defibrotide in 54 cases. Other VOD/SOS-targeted treatments mentioned in single cases were corticosteroids (2 patients), acetylcysteine, ursodeoxycholic acid, eculizumab, low molecular weight heparin, and transjugular intrahepatic portosystemic shunt.

Three patients with severe and four with very severe VOD/SOS were not given VOD/SOS- targeted treatment.

The severity grade did not affect the VOD/SOS-targeted treatment. Defibrotide was given to 3/3 patients with moderate, 23/26 with severe and 28/31 patients with very severe VOD/SOS.

Without the modification of grade based on risk factors, 3 patients would have had mild and 13 moderate grade VOD/SOS. Defibrotide treatment was given to all these patients with mild and 12/13 patients with moderate grade.

### Resolution of VOD/SOS

Resolution was defined as maintained normalization of the symptoms and signs of VOD/SOS. The complication resolved in 37 of 64 patients (58%), in 3/3 with moderate, 22/28 (79%) with severe, and 12/33 (36%) with very severe VOD/SOS (*p* < 0.001). The information was lacking of one patient with severe and two with very severe VOD/SOS.

If the effect of risk factors is omitted, VOD/SOS resolved in 3/3 patients with mild, 10/12 with moderate, 12/16 with severe, and 12/33 with very severe grade complication.

### Survival

**Fig. 2:**
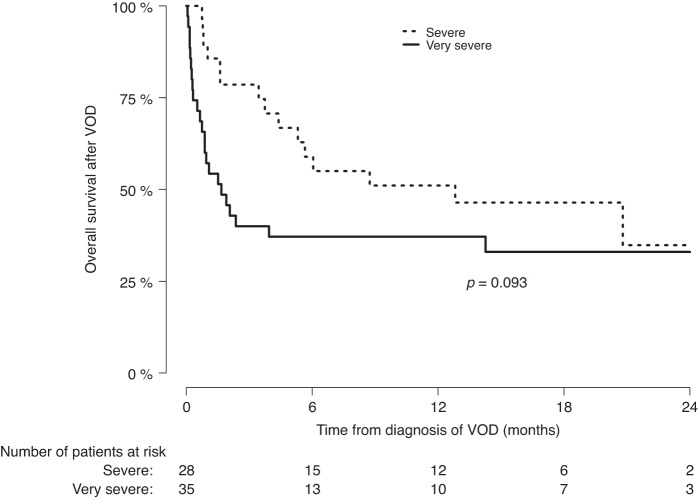
Overall survival of patients with severe and very severe VOD/SOS.

The median follow-up of the VOD/SOS patients from the transplantation was 19.1 (95% CI 15.5–21.0) months. Thirty-eight of the 67 patients (57%) survived by day 100 post transplantation, 3/3 in moderate, 22/29 (76%) in severe, and 13/35 (37%) in very severe VOD/SOS (*p* = 0.002). The survival of patients with severe vs. very severe grade VOD/SOS is shown in Fig. 2. The survival at six months was 58.9% and 37.1% (*p* = 0.093) in the severe and very severe grade, respectively.

Of 40 deceased patients, the causes of death were reported in 36 cases. All deaths were due to non-relapse causes. VOD/SOS was listed among the causes of death in 20, MOD/MOF in 21 cases.

If the effect of risk factors is omitted, the survival by day 100 according to the severity grade was 3/3 in mild, 11/13 in moderate, 11/16 in severe, and 13/35 in very severe VOD/SOS.

## Discussion

The incidence of VOD/SOS observed in the present study, cumulative incidence at 21 days 1.8% and at 100 days 2.4%, was low compared with previous reports [1, 2, 4–7]. The reported incidences have been variable due to differences in patient populations, prior therapies, conditioning regimens, types and methods of transplantation, and criteria used. The criteria have a significant impact on the incidence. Of the two classical sets of criteria, the Seattle criteria are less stringent and produce a higher incidence than that seen with the Baltimore criteria [2] or the EBMT criteria largely based on the latter criteria. Another set of criteria was recently presented by Cairo et al.[7] to identify patients who may be missed by using traditional diagnostic criteria. The incidence figures of the present study were calculated from the center reports, including all patients diagnosed with VOD/SOS at the center, regardless of the criteria used. Detailed information of the VOD/SOS cases was available of only approximately 75% of the cases, and in some instances the stringent EBMT criteria, applied in this study, had not been followed. Therefore, with the EBMT criteria the incidence would have been even slightly lower. However, the possibility of under-reporting of this complication has to be considered. Bazarbachi et al.[12] studied 202 allogeneic transplant patients reported to have died of MOF. They found that VOD/SOS-related MOF was widely under-reported, accounding for 27% of deaths attributed to MOF of unknown origin without a previous VOD/SOS diagnosis.

The present findings suggest that the incidence of VOD/SOS has decreased in the recent years, as proposed previously [3]. This is likely to be multifactorial and be based on developments in the transplantation methods and possibly patient selection. Conditioning is thought to play a central role as a cause of VOD/SOS, and it is therefore of interest that the patients with VOD/SOS had not received more often MAC than the patients who had not developed this complication. A similar observation has been made by Bazarbachi et al.[12]. A possible cause for the reduced incidence might be wider use of VOD/SOS prophylaxis at the present time compared with earlier years, but data to support this is not readily available.

The EBMT classification presented new detailed criteria for late-onset VOD/SOS. The proportion of late-onset cases of all VOD/SOS in the present material was 22% which is in line with previous reports [7, 13–16], although higher proportions have been presented [13, 16]. There are three different sets of criteria for the diagnosis of the late-onset form, but it turned out that in the present relatively small group of 15 patients the diagnosis could be made with the criteria of classical VOD/SOS occurring after day 21 in all patients with only one exception.

Approximately half of the patients had received VOD/SOS prophylaxis. The policies were variable, the most common agent used was ursodeoxycholic acid. A large majority received treatment with defibrotide, the only drug approved for the treatment of VOD/SOS. The complication resolved in 58% of the cases, and 57% of the patients were alive at day 100. These are findings similar to previous reports of patients treated with defibrotide [5, 17–19].

In the present material, the EBMT classification distributed most of the cases into the severe and very severe grade. There were no mild cases and only three moderate cases (4%). A similar trend, although not quite as strong, was seen in the study of Yoon et al. [20]. In their patient material diagnosed according to the EBMT criteria, the proportions of mild and moderate severity grade were 3.2 and 9.6%, respectively. Generally, it is possible that mild cases of complications, such as VOD/SOS, are reported less actively than more severe cases.

In the EBMT severity grading the role of risk factors is essential. According to the classification, mild cases with two or more risk factors must be graded as moderate, and similarly moderate cases with two or more risk factors as severe. As almost all of the present patients (65/67) had at least two risk factors, this had a major effect on the proportion of the lower grades. If the impact of risk factors was disregarded, the distribution became more even. However, we believe that given the dismal outcome of VOD/SOS, such conservative approach is useful to increase awareness, and in order to facilitate early diagnosis and intervention.

The prognostic value of the severity grades could only be assessed in the severe and very severe grades; the survival was worse in the very severe grade. The mild-moderate cases were too few to be analyzed. If the impact of risk factors was disregarded, the survival worsened grade by grade from mild to very severe grade, but the differences were not significant. This is possibly due to small patient numbers in the lower grades. With one exception, all patients with very severe VOD/SOS had MOD/MOF, and therefore MOD/MOF was the central prognostic factor for survival.

The representativeness of the findings of the present study for the situation at EBMT centers is likely to be rather good. Seventy-one centers from 20 countries with a total of more than 2800 patients participated. However, the incidence of VOD/SOS was low and detailed description of the complication was lacking from one quarter of the patients, and therefore the absolute numbers of cases available for the detailed analysis were relatively low. This has to be taken into account when interpreting the results, as well as possible under-reporting which is increasingly recognized [12].

In the present study we observed a lower incidence of VOD/SOS compared to old historical data which indicates that the incidence has most probably declined in the recent years, likely due to safer transplant procedures, improved awareness, mitigation of risk factors, and better prophylaxis. However, VOD/SOS is still a serious complication; by day 100 post transplantation more than 40% of the patiens had died. The impact of risk factors for the severity grading was strong, further emphasizing the need to continue to refine the impact of individual components of the severity assessment in the definitions of the severity grades.

### Supplementary information

Supplementary Table 1

Supplementary Table 2
