# Supplementary material for: Current incidence, severity, and management of veno-occlusive disease/sinusoidal obstruction syndrome in adult allogeneic HSCT recipients: an EBMT Transplant Complications Working Party study
Source: Bone Marrow Transplant. 2023 Aug 12;58(11):1209–14. doi: 10.1038/s41409-023-02077-2 (PMC10622315; doi:10.1038/s41409-023-02077-2)
Supplement: Supplementary file 1 — Supplementary Table 1 [file 41409_2023_2077_MOESM1_ESM.pdf]

Supplementary Table 1. Definition of multiorgan dysfunction/failure

In addition to VOD/SOS, one or more of the following:

Renal: creatinine ≥2 times the level at admission for conditioning or ≥2 times the lowest level during conditioning, or creatinine clearance or glomerular filtration rate ≤50% the level at admission, or dialysis dependence.

Pulmonary: oxygenation saturation ≤90% on room air and/or the need for positive pressure/ventilator dependence not attributable to any other cause.

Central nervous system: confusion, lethargy, and/or delirium not attributable to any other cause.
