# Supplementary material for: Current incidence, severity, and management of veno-occlusive disease/sinusoidal obstruction syndrome in adult allogeneic HSCT recipients: an EBMT Transplant Complications Working Party study
Source: Bone Marrow Transplant. 2023 Aug 12;58(11):1209–14. doi: 10.1038/s41409-023-02077-2 (PMC10622315; doi:10.1038/s41409-023-02077-2)
Supplement: Supplementary file 2 — Supplementary Table 2 [file 41409_2023_2077_MOESM2_ESM.pdf]

Supplementary Table 2. Participating centers

CIC	Physician	City	Country
993	Nabil Yafour	Oran	Algeria
234	Xavier Poiré	Brussels	Belgium
630	Rik Schots	Brussels	Belgium
209	Johan Maertens	Leuven	Belgium
726	Yves Beguin	Liege	Belgium
211	Yana Novis	Sao Paulo	Brasil
597	Jiri Mayer	Brno	Czech Republic
574	Ludek Raida	Olomouc	Czech Republic
718	Pavel Jindra	Pilsen	Czech Republic
515	Urpu Salmenniemi	Helsinki	Finland
225	Maija Itälä-Remes	Turku	Finland
650	Mathilde Hunault-Berger	Angers	France
251	Gandhi Damaj	Caen	France
270	Claude Eric Bulabois	Grenoble	France
671	Hélenè Labussière-Wallet	Lyon	France
926	Nathalie Fegueux	Montpellier	France
523	Thomas Cluzeau	Nice	France
941	Herve Tilly	Rouen	France
250	Denis Guyotat	Saint Etienne	France
672	Bruno Lioure	Strasbourg	France
666	Jean Henri Bourhis	Villejuif	France
259	Thomas Schroeder	Essen	Germany
552	Gerald G. Wulf	Goettingen	Germany
295	Arnold Ganser	Hannover	Germany
524	Peter Dreger	Heidelberg	Germany
533	Inken Hilgendorf	Jena	Germany
256	Thomas Valerius	Kiel	Germany

145	Martin Kaufmann	Stuttgart	Germany
561	Ioanna Sakellari	Thessaloniki	Greece
345	Tsila Zuckerman	Haifa	Israel
658	Alessandro Rambaldi	Bergamo	Italy
141	Domenico Russo	Brescia	Italy
354	Stefania Bramanti	Milano	Italy
544	Matteo Parma	Monza	Italy
285	Alessandra Biffi	Padova	Italy
1006	Marco Zecca	Pavia	Italy
795	Mario Petrini	Pisa	Italy
587	Massimo Martino	Reggio Calabria	Italy
287	Luigi Rigacci	Rome	Italy
307	Simona Sica	Rome	Italy
756	William Arcese	Rome	Italy
705	Renato Fanin	Udine	Italy
502	Cristina Skert	Venezia	Italy
797	Marco Ruggeri	Vicenza	Italy
369	Ali Bazarbachi	Beirut	Lebanon
677	Grzegorz Helbig	Katowice	Poland
693	Kazimierz Halaburda	Warsaw	Poland
725	Aleksandr Kulagin	St. Petersburg	Russia
858	Mohamed Bayoumy	Jeddah	Saudi Arabia
434	Aloysius Ho	Singapore	Singapore
613	Josep Maria Ribera Santasusana	Badalona	Spain
214	Montserrat Rovira	Barcelona	Spain
238	Concepción Herrera Arroyo	Cordoba	Spain
309	José Luis López Lorenzo	Madrid	Spain
819	J. L. Diez-Martin	Madrid	Spain
323	José Maria Moraleda	Murcia	Spain
737	Jose Rifón	Pamplona	Spain
727	Dolores Caballero	Salamanca	Spain

242	Arancha Bermúdez Rodriguez	Santander	Spain
769	José Antonio Pérez-Simón	Sevilla	Spain
202	Jakob Passweg	Basel	Switzerland
237	N. Schaap	Nijmegen	The Netherlands
246	Annoek E. C. Broers	Rotterdam	The Netherlands
589	Hakan Ozdogu	Adana	Turkey
169	Zubeyde Nur Ozkurt	Ankara	Turkey
617	Gunhan Gurman	Ankara	Turkey
911	Akif Yesilipek	Antalya	Turkey
943	Ümit Barbaros Üre	Istanbul	Turkey
994	Mutlu Arat	Istanbul	Turkey
303	Keith M. O. Wilson	Cardiff	United Kingdom
778	John Snowden	Sheffield	United Kingdom
